# Progress in Introduction of Pneumococcal Conjugate Vaccine — Worldwide, 2000–2012

**Published:** 2013-04-26

**Authors:** Susan A. Wang, Carsten F. Mantel, Marta Gacic-Dobo, Laure Dumolard, Thomas Cherian, Brendan Flannery, Jennifer D. Loo, Jennifer R. Verani, Cynthia G. Whitney

**Affiliations:** Dept of Immunizations, Vaccines, and Biologicals, World Health Organization, Geneva, Switzerland; Global Immunization Div, Center for Global Health; Div of Bacterial Diseases, National Center for Immunization and Respiratory Diseases, CDC

Pneumococcal conjugate vaccines (PCVs) are safe and effective for reducing illness and deaths caused by *Streptococcus pneumoniae* (1). Recommendations for PCV use from the World Health Organization (WHO) (1,2) and funding from the GAVI Alliance have resulted in an increase in PCV introductions into national immunization programs, especially in lower-income countries. Additionally, new formulations that cover more serotypes commonly causing disease in lower- and middle-income countries have become available. This report uses WHO data from 2000–2012, stratified by country disease burden characteristics and World Bank country income groups, to describe global progress in PCV introduction. As of December 2012, a total of 86 (44%) WHO member states have added PCV to the routine infant immunization schedule of their national immunization programs; among those, 23 have introduced PCV with GAVI Alliance support. PCV introduction among WHO member states was most common in the Americas Region (60% of member states), followed by the Eastern Mediterranean Region (50%), European Region (49%), African Region (41%), and Western Pacific Region (33%); none of 11 WHO member states in the South-East Asia Region have introduced PCV. Proportions of low- and middle-income countries with PCV introductions were similar. The proportion of the world’s birth cohort living in countries with PCV in national immunization programs increased from 1% in 2000 to 31% in 2012. These findings suggest that efforts to increase PCV introduction and use globally are succeeding; however, gaps in PCV use remain in Asia and countries with large birth cohorts, where concerted efforts should be focused.

Worldwide, an estimated 14.5 million episodes of serious pneumococcal disease (including pneumonia, meningitis, and sepsis) occur each year in children aged <5 years, resulting in approximately 500,000 deaths, almost all of which occur in low- and middle-income countries (3). PCV was first licensed in 2000 as a formulation that provided protection against seven of the most common pneumococcal serotypes. In 2006, WHO recommended that PCV be included in all routine immunization programs, especially in countries with high pneumococcal disease burden, defined as >10% of deaths in children aged <5 years attributed to pneumonia or pneumonia mortality rate of >50 deaths per 1,000 live births among children aged <5 years (1). Beginning in 2010, new PCV formulations protecting against 10 and 13 serotypes have become available for use, offering better coverage for serotypes commonly causing disease in low- and middle-income countries (4).

To assess the status of global PCV introduction, a WHO database tracking vaccine introductions was used to identify countries that included PCV in routine infant immunization schedules as of December 2012. PCV introductions were characterized by WHO region (5), eligibility for GAVI Alliance financial support,* World Bank income classification,† and pneumonia disease burden (6). The proportion of the global birth cohort living in countries that had introduced PCV was calculated using United Nations 2010 birth cohort estimates (6). For countries that introduced PCV during 2000–2009, WHO–United Nations Children’s Fund (UNICEF) estimated coverage for the full 3-dose series of PCV was used, if available (5). Operational issues related to PCV introduction were identified through WHO post-introduction evaluations.

As of December 2012, 86 (44%) of 194 WHO member states had introduced PCV into national immunization programs, up from one (1%) in 2000 (Figure 1), representing 31% of all children born in WHO member states. PCV was introduced in national immunization programs in 21 (60%) of 35 member states in the Americas Region, 11 (50%) of 22 member states in the Eastern Mediterranean Region, 26 (49%) of 53 member states in the European Region, 19 (41%) of 46 member states in the African Region, nine (33%) of 27 member states in the Western Pacific Region, and none of 11 member states in the South-East Asia Region (Figure 2). By income level, 36 (73%) of 50 high-income countries introduced PCV; proportions were lower for remaining income strata: 13 (37%) of 36 low-income, 18 (35%) of 52 lower-middle income, and 18 (34%) of 53 upper-middle income countries. Among 72 countries that were eligible for phase II (2007–2010) financial support from the GAVI Alliance for PCV introduction, 23 (32%) introduced PCV and all low-income introductions occurred with GAVI Alliance support. By 2012, 21 (36%) of 59 high-mortality countries and 38 (37%) of 102 countries in which >10% of deaths in children aged <5 years were attributable to pneumonia had introduced PCV. Among countries using PCV, coverage with 3 doses of PCV assessed at age 12–24 months was highest among high- and low-income countries (median: 92% and 95%, respectively); less among upper-middle income countries (median: 76%), and lowest among lower-middle income countries (median: 44%) (Table).

Reports from 11 postintroduction evaluations conducted during 2010–2012 in low- and middle-income African countries described programmatic issues related to PCV introductions. First, programs need accurate data to define target populations, accompanied by clear messages to health workers and the community to prioritize target populations. In several countries, children outside the target infant age group also were brought for vaccination and, in most cases, were vaccinated, creating shortages of PCV for the target age group. Second, health worker knowledge that PCV provides protection against only one cause of pneumonia was crucial to ensure that they educated caretakers about other options for prevention and treatment of pneumonia. Third, weaknesses were identified in the underlying capacity of the immunization systems, including needs for 1) innovative training approaches to address the complexity of messages relating to use of new vaccines, 2) improvement of supportive supervision, and 3) further enhancement of injection safety, injection waste management, and monitoring of adverse events after immunization.

## Editorial Note

Use of PCV has increased substantially since 2000, especially in low- and middle- income countries, where the burden of pneumococcal disease and deaths is high. A critical factor enabling vaccine introductions in low-income countries has been support from the GAVI Alliance. As of the first quarter of 2013, 24 countries have introduced PCV with GAVI Alliance support, and 27 additional countries have been approved for GAVI Alliance–supported PCV introductions (7). However, important gaps in PCV introduction remain, notably in the WHO South-East Asia Region and in countries with large birth cohorts. The lack of PCV introduction in several large countries is reflected in the gap between the proportion of countries having introduced PCV (44%) and the proportion of the world’s birth cohort living in countries that have introduced PCV (31%). Low- and middle-income countries lagged behind high-income countries, with all low-income introductions attributed to GAVI Alliance support. Middle-income countries are not eligible for GAVI Alliance support and need to weigh vaccine procurement and operational costs against costs of other health priorities. Additionally, only two companies (Pfizer and GlaxoSmithKline) manufacture any of the three PCV formulations; each formulation is supplied by one company and insufficient supply has led to delays in planned introductions in some countries. Although pneumonia is a leading killer of children in the majority of countries, the disease burden preventable by PCV might be unrecognized, decreasing local demand for the vaccine. Improved data on the impact of PCV vaccination in reducing and preventing disease caused by *Streptococcus pneumoniae* will help guide policy decisions about PCV introduction and sustained use (1).

The findings of this report are subject to at least two limitations. First, the vaccination coverage estimates might differ from actual coverage because of inaccurate reporting of target population size or number of doses administered. Additionally, coverage estimates were only from countries using PCV for at least 2 years and might not be reflective of coverage achieved in all settings.

In spite of these challenges, the rate at which PCV has been introduced into childhood immunization programs worldwide has been faster than that of other new vaccines in the past (8). In addition to WHO recommendations and GAVI Alliance support, other measures are encouraging PCV use around the world. WHO and UNICEF promote PCV use in the integrated *Global Action Plan for Pneumonia and Diarrhoea (GAPPD)* as a comprehensive approach to reducing pneumonia morbidity and mortality and as an important strategy for achieving United Nations Millennium Development Goal 4 to reduce child mortality (9). Further information on the magnitude of PCV benefits in low- and middle-income countries might encourage more policy makers to introduce PCV into immunization programs; studies are ongoing and data on the impact and effectiveness of PCV in reducing disease caused by *Streptococcus pneumoniae* in these settings is being prepared for publication. A WHO manual for measuring the impact of the *Haemophilus influenzae* type b conjugate vaccine and PCV can assist with designing studies to determine how PCV performs in a variety of settings.§ The progress of PCV introductions noted in this report and anticipated PCV introductions in coming years will help reduce the burden of pneumonia and pneumococcal disease worldwide.

What is already known on this topic?

Globally*, Streptococcus pneumoniae* is a significant cause of pneumonia, meningitis, and sepsis in children aged <5 years and leads to an estimated 14.5 million episodes of serious disease and approximately 500,000 deaths annually. Pneumococcal conjugate vaccines (PCVs) are safe and effective for prevention of this disease, and the World Health Organization (WHO) recommends that PCV be included in all routine immunization programs.

What is added by this report?

PCV use increased from one (1%) WHO member state in 2000 to 86 (44%) in 2012. Gaps in PCV introductions were noted in Asia and in countries with large birth cohorts; only 31% of the world’s birth cohort currently has access to PCV. WHO recommendations for use, financial support through the GAVI Alliance for PCV introduction in lower-income countries, and newer PCV formulations that protect against additional serotypes likely contributed to the increased use of PCV.

What are the implications for public health practice?

Increased use of PCV will help reduce the incidence of pneumonia and pneumococcal disease worldwide. The success of PCV introductions and the lessons learned from countries that have added PCV to their immunization programs will help guide decisions about future PCV introductions and sustained use.

## Figures and Tables

**FIGURE 1:**
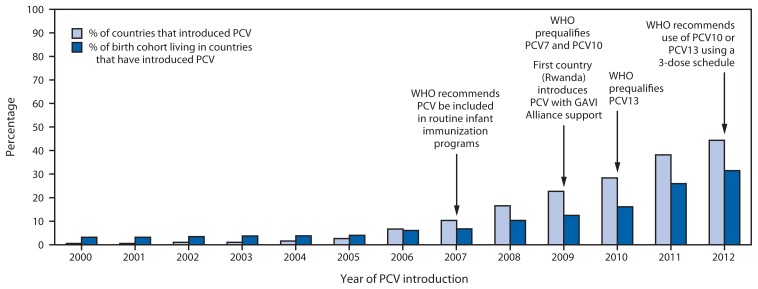
Progress of pneumococcal conjugate vaccine (PCV) introductions and proportions of birth cohorts living in countries that have introduced PCV into routine infant immunization schedules, by year — World Health Organization (WHO), worldwide, 2000–2012 **Abbreviations:** PCV7 = 7-valent PCV; PCV10 = 10-valent PCV; PCV13 = 13-valent PCV.

**FIGURE 2:**
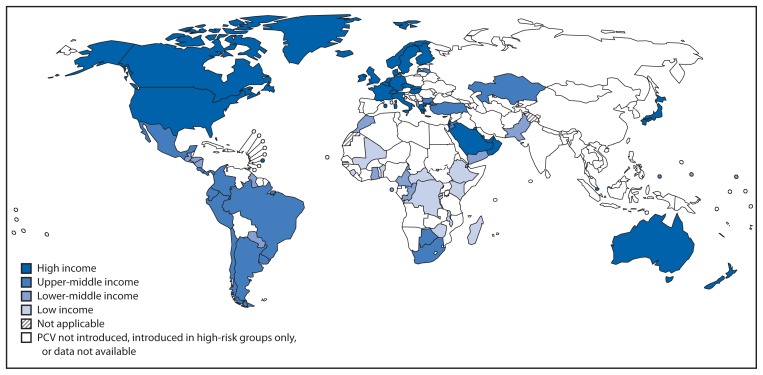
Countries that have introduced pneumococcal conjugate vaccines in their national Immunization programs, by income status^*^ — worldwide, 2012 **Data sources:** World Health Organization/Immunization Vaccines and Biologicals/Expanded Programme on Immunization 2013 database, and World Bank list of economies (July 2012). ^*^ World Bank income groups are defined (in U.S. dollars) as follows: high-income countries = countries with a 2011 gross national income (GNI) per capita ≥$12,476; upper-middle income countries = countries with a 2011 GNI <$12,476 and ≥$4,036; lower-middle income countries = countries with a 2011 GNI <$4,035 and ≥$1,027; and low-income countries = countries with a 2011 GNI ≤$1,026. No income status was reported for Niue. Additional information is available at http://data.worldbank.org/indicator/ny.gnp.pcap.cd.

**TABLE:** Numbers of countries with pneumococcal conjugate vaccine (PCV) introductions and PCV coverage, by World Bank income group* and characteristics associated with high burden of pneumococcal disease — worldwide, 2012

PCV introduction status/coverage	High income (n = 50)		Upper-middle income (n = 53)		Lower-middle income (n = 52)		Low income (n = 36)		No income status† (n = 3)		Total (N = 194)	
					
	No.	(%)	No.	(%)	No.	(%)	No.	(%)	No.	(%)	No.	(%)
**Number of countries with PCV introductions**												
Added PCV to the routine infant immunization schedule	36	(73)	18	(34)	18	(35)	13	(37)	1	(33)	**86**	**(44)**
Offering PCV for high-risk populations only	3	(6)	2	(4)	0	—	0	—	0	—	**5**	**(3)**
No PCV introduction to date	11	(22)	33	(62)	34	(65)	23	(64)	2	(67)	**103**	**(53)**
**Number of countries with PCV introductions among countries with high burden of pneumococcal disease**												
Phase II GAVI Alliance–eligible countries	0	—	3	(6)	33	(63)	36	(100)	0	—	**72**	**(38)**
PCV introductions with GAVI Alliance support	NA	—	0	—	10	(30)	13	(36)	NA	—	**23**	**(32)**
Countries with mortality >50 per 1,000 live births among children aged <5 years (i.e., high child mortality rate)	1	(2)	4	(8)	22	(42)	32	(89)	0	—	**59**	**(31)**
PCV introductions in high child mortality rate countries	0	—	1	(25)	7	(32)	13	(41)	NA	—	**21**	**(36)**
Countries with >10% deaths attributed to pneumonia among children <5 years	2	(4)	20	(38)	43	(83)	35	(97)	2	(67)	**102**	**(53)**
PCV introductions in countries with high rates of child pneumonia deaths	1	(50)	7	(35)	16	(37)	13	(37)	1	(50)	**38**	**(37)**
**PCV coverage**												
Number of countries reporting 2011 coverage for 3 doses of PCV	21		12		4		2		1		**40**	
Median coverage for 3 doses of PCV in 2011		(92)		(76)		(44)		(95)		(99)		**(90)**
Range		(1–99)		(46–98)		(23–67)		(93–97)				**(1–99)**

**Abbreviation:** NA = not applicable.

* World Bank income groups are defined (in U.S. dollars) as follows: high-income countries = countries with a 2011 gross national income (GNI) per capita ≥$12,476; upper-middle income countries = countries with a 2011 GNI <$12,476 and ≥$4,036; lower-middle income countries = countries with a 2011 GNI <$4,035 and ≥$1,027; and low-income countries = countries with a 2011 GNI ≤$1,026. Additional information is available at http://data.worldbank.org/indicator/ny.gnp.pcap.cd.

† Countries with no income status: Cook Islands, Nauru, and Niue.

## Footnotes

* Eligibility for phase II GAVI Alliance support (2007–2010) was based on having a gross national income (GNI), in U.S. dollars, of ≤$1,000 in 2003 and diphtheria-tetanus-pertussis vaccination coverage >50%.

† World Bank income groups, in U.S. dollars, are defined as follows: high-income countries: countries with a 2011 GNI per capita ≥$12,476; upper-middle income countries: countries with a 2011 GNI <$12,476 and ≥$4,036; lower-middle income countries: countries with a 2011 GNI <$4,035 and ≥$1,027; and low-income countries: countries with a 2011 GNI ≤$1,026. Countries with no income status: Cook Islands, Nauru, Niue. Additional information is available at http://data.worldbank.org/indicator/ny.gnp.pcap.cd.

§ Available at http://www.who.int/immunization/documents/WHO_IVB_12.08/en/index.html.
